# High Index Values of Enzyme-Linked Immunosorbent Assay for BP180 at Baseline Predict Relapse in Patients With Bullous Pemphigoid

**DOI:** 10.3389/fmed.2018.00139

**Published:** 2018-05-09

**Authors:** Hiroshi Koga, Kwesi Teye, Norito Ishii, Chika Ohata, Takekuni Nakama

**Affiliations:** ^1^Department of Dermatology, Kurume University School of Medicine, Fukuoka, Japan; ^2^Kurume University Institute of Cutaneous Cell Biology, Fukuoka, Japan

**Keywords:** bullous pemphigoid, BP180 ELISA, BP230 ELISA, eosinophil, IgE, predictive marker, relapse

## Abstract

Bullous pemphigoid (BP) presenting with erythema plaques and tense blisters is the most frequent autoimmune bullous disease. Immunologically, BP is characterized by the presence of circulating anti-epidermal basement membrane zone (BMZ) antibodies. The autoantigens in BMZs targeted by patient's antibodies are mainly BP180 (type XVII collagen) and BP230. Previous reports have indicated that IgG to the immunodominant region of BP180 in BP, 16th non-collagenous domain (NC16A), and anti-BP180NC16A IgE are related to disease activity. In the cytokine profile, serum levels of IL-6, TNF-α, IL-15, and CCL18 were associated with the severity or activity of the disease. Blood eosinophilia is seen frequently, especially in severe cases. These biomarkers are helpful to evaluate efficacy of treatment and disease severity. Due to the high frequency of disease relapse, prediction of relapse at initiation of treatment (baseline) must be beneficial for clinicians. Therefore, we evaluated biomarkers anti-BP180 IgG (BP180 ELISA), anti-BP230 IgG (BP230 ELISA), peripheral eosinophils, and serum IgE at baseline between BP patients with (*n* = 16) and without (*n* = 31) relapse. We found significantly higher index values of BP180 ELISA in the relapse group, whereas no significant difference was found in BP230 ELISA, peripheral eosinophils, and serum IgE. This study indicated that a high index value of BP180 ELISA (cutoff value, 53.09 U/mL; sensitivity, 81.3%; specificity, 48.4%) at baseline may predict relapse in patients with BP. This may help clinicians treating BP patients in decision-making regarding duration and intensity of treatment.

## Introduction

Bullous pemphigoid (BP) presents with erythema plaques and tense blisters, and is one of the pemphigoid diseases occurring most frequently among autoimmune bullous diseases, with an especially high prevalence in the elderly. Immunologically, BP is characterized by presence of circulating anti-epidermal basement membrane zone (BMZ) antibodies, which are detected by indirect immunofluorescence (IF), and antibodies and/or complement deposition on BMZ, which is detected by direct IF. The autoantigens in BMZ targeted by patient's antibodies are mainly BP180 (type XVII collagen) and BP230 (1). The immunodominant region of BP180 in BP is the 16th non-collagenous domain (NC16A) and anti-BP180NC16A IgG is related to disease activity (2), whereas anti-BP230 is not (3). Total serum IgE level is elevated in BP and anti-BP180NC16A IgE recently was reported to be associated with disease activity (4, 5). In the cytokine profile, serum levels of interleukin (IL)-5, IL-6, and IL-8 were elevated in BP and serum levels of IL-6, tumor necrosis factor (TNF)-α, IL-15 and chemokine (C-C motif) ligand 18 (CCL18) were associated with severity or disease activity (6–9). Blood eosinophilia is seen frequently, especially in severe cases (10). These biomarkers are helpful to evaluate efficacy of treatment and disease severity.

In terms of treatment, dapsone, immunosuppressants, such as azathioprine, cyclosporine, and methotrexate, and emerging therapies, including high-dose intravenous immunoglobulins (IVIG), immunoadsorption, rituximab, and omalizumab, are available, although systemic corticosteroid remains in main strategy for treatment (11). However, previous studies reported disease relapse frequently ranging from 29.2 to 39% during the first year (12, 13). To date, several studies have reported predictive factors of relapse, including BP180 enzyme-linked immunosorbent assay (ELISA) titer > 23 U/mL 150 days later at baseline (13) and a positive finding on direct IF and BP180 ELISA titer > 27 U/mL at cessation of therapy (14).

Prediction of relapse at initiation of treatment (baseline) must be more beneficial for clinicians. Therefore, extensive disease activity, defined as the occurrence of at least 10 new blisters daily, and association with dementia were found to be risk factors for relapse in multivariable analysis (14). However, prediction of relapse by biomarker at baseline in BP has not yet been fully analyzed. We evaluated biomarkers at baseline previously reported as being associated with disease activity in BP patients with and without relapse.

## Materials and methods

### Patients

Patients with BP who visited the Kurume University Hospital between October 2005 and May 2016 and were followed at our hospital for more than 1 year were enrolled in this study. To avoid treatment bias, patients treated with systemic corticosteroid and/or immunosuppressants at first visit to our hospital were excluded. A total of 47 patients were analyzed. Patients with BP were diagnosed based on the combination of clinical, histopathological, and immunological findings of circulating IgG reaction on the roof of 1 M NaCl-split-normal human skin, and/or positive finding on BP180 ELISA. Relapse was defined in this study when the dose of prednisolone was increased 1.5-fold (former dose >10 mg/day) or over 10 mg/day (former dose <10 mg/day) after the consolidation phase defined by Murrell et al. (15). Information on treatment and the number of peripheral eosinophils was obtained from the medical record. Regarding treatments, the present cases were primarily treated with systemic and topical corticosteroids. The intractable cases were treated with an additional immunosuppressant, pulse corticosteroid therapy with methylprednisolone, and double-filtration plasmapheresis. Only one case in non-relapse group was treated with IVIG. No patient was treated with either rituximab or omalizumab.

### Enzyme-linked immunosorbent assay (ELISA)

Circulating IgG to BP180NC16A and BP230 were detected using commercial kits (MESACUP BP180 and BP230 ELISA kits; MBL Co., Nagoya, Japan) and were used according to the manufacturer's instructions. Further dilution to 1:1,600 and/or 1:16,000 was performed for samples with an index >100 with 1:101 dilutions to obtain reliable index values (16–18). For total serum IgE, a human IgE ELISA quantification set (Bethyl Laboratories, Montgomery, TX, USA) was used according to the manufacturer's instructions.

### Immunofluorescence (IF)

Direct IF was performed using perilesional skin specimens from each patient as described previously (19). Indirect IF using 1 M NaCl-split-normal human skin was performed as described previously (20, 21).

### Receiver operating characteristic (ROC) analysis

ROC analysis was performed on indexes of BP180 ELISA to assess predictive accuracy for relapse (ROC-AUC 0.68). The highest Youden Index (0.40) set the cutoff value at 90.07 U/mL, with 75.0% sensitivity and 64.5% specificity.

### Statistical analysis

Data were presented as mean ± SD. Statistical analysis of age, BP180 ELISA index, BP230 ELISA index, peripheral eosinophils, and serum IgE at baseline in the two groups was performed using a two-tailed Mann-Whitney *U*-test, and sex in the two groups was analyzed using the Fisher's exact test using GraphPad Prism (Version 6.05; GraphPad Software, San Diego, CA). A *P-*value of 0.05 was considered statistically significant.

## Results

In our study, 34% of the patients experienced relapse. The latency to relapse was 367 ± 285 days; 10 were in the first year, 5 were in the second year, and 1 was in the fourth year. BP180 ELISA indexes at baseline were 1138 ± 2161 U/mL, 459 ± 334 U/mL, and 375.2 U/mL, respectively. In the 16 patients with relapse, 4 were on “complete remission off therapy,” 10 were on “Minimal therapy,” and 2 were on “treatment with more than 0.1 mg/kg/day of prednisolone” before relapse.

**Figure 1:**
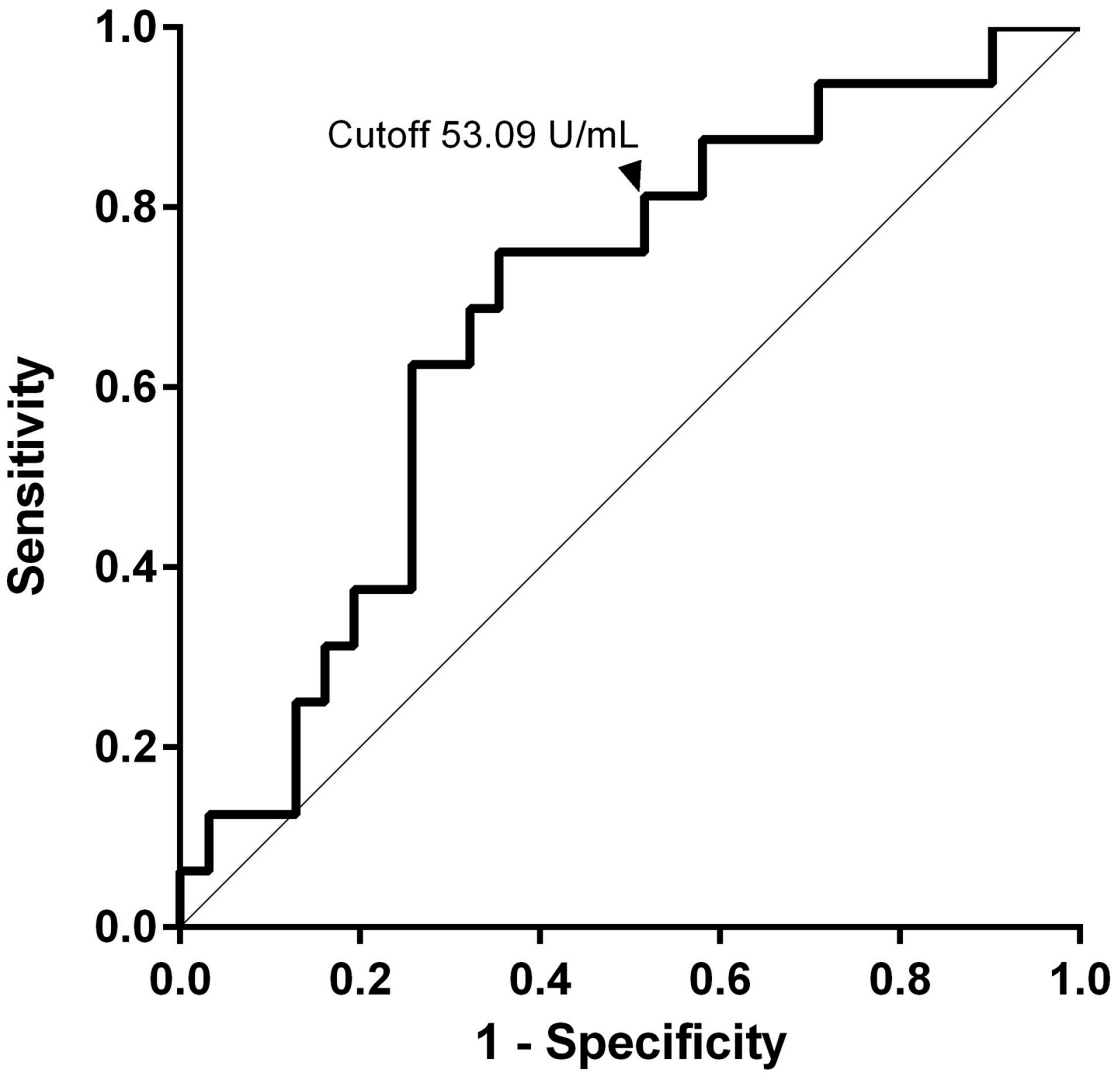
Values of anti-BP180NC16A IgG at baseline for prediction of relapse. The ROC curve was used to evaluate the ability of BP180 ELISA index at baseline to predict relapse. The cutoff value was set at 53.09 U/ml with 81.3% sensitivity and 48.4% specificity.

We measured BP180 and BP230 ELISA indexes, and total serum IgE levels using patients' sera at baseline and compared the results in the two groups of patients with or without relapse (Table 1). Of note, BP180 ELISA index was significantly higher in the relapse group (877.8 ± 1718 U/mL) than in the non-relapse group (381.2 ± 959.9 U/mL). On the other hand, sex ratio, age, BP230 ELISA index, peripheral eosinophils, and total serum IgE were not significantly different between the two groups, although serum IgE level was higher in the relapse group (13,350 ± 20,887 ng/mL) than in the non-relapse group (6614 ± 9355 ng/mL) (*p* = 0.176). To determine the cutoff value for prediction of relapse, a receiver operating characteristic (ROC) curve was obtained from BP180 ELISA indexes at baseline (ROC-AUC 0.68). The cutoff value set by the Youden Index was 90.07 U/mL, with 75.0% sensitivity and 64.5% specificity. To reduce false-negatives, we set the cutoff value at 53.09 U/ml with 81.3% sensitivity and 48.4% specificity (Figure 1). We also checked positive reaction to BP180 and BP230 by ELISA in our cases. The positive rates to BP180, BP230, and both combined were similar between the two groups (Table 1). However, of note, all three cases with negative reactivity in both ELISAs were from the non-relapse group, and none from the relapse group.

**Table 1:** Characteristics of BP patients at baseline.

	**Total (*n* = 47)**	**Non-relapse (*n* = 31)**	**Relapse (*n* = 16)**	***p*-value**
Sex ratio (female/male)	1.35	1.21	1.67	0.758
Age (years)	74.68 ± 13.00	73.55 ± 10.83	76.88 ± 16.59	0.157
BP180 ELISA index (U/mL)	550.3 ± 1273	381.2 ± 959.9	877.8 ± 1718	0.041
BP230 ELISA index (U/mL)	144.9 ± 283.3	145.7 ± 321.3	143.4 ± 199.2	0.303
Peripheral eosinophils (/μL)	1002 ± 1010	848.0 ± 872.9	1320 ± 1218	0.135
Serum IgE (ng/mL)	8907 ± 14483	6614 ± 9355	13350 ± 20887	0.176
**POSITIVITIES To BP180/BP230 ELISA**				
BP180 alone (n)	13 (27.7%)	8 (25.8%)	5 (31.3%)	
BP230 alone (n)	3 (6.4%)	2 (6.5%)	1 (6.3%)	
Double positive (n)	28 (59.6%)	18 (58.1%)	10 (62.5%)	
Double negative (n)	3 (6.4%)	3 (9.7%)	0 (0.0%)	

## Discussion

We showed that the concentration of anti-BP180NC16A IgG antibodies in serum at baseline measured by BP180 ELISA was significantly higher in the relapse group than in the non-relapse group. This result suggested that a high BP180 ELISA index before starting treatment is a predictive marker for clinical relapse in later stages of BP. Fichel et al. (13) did not reveal significant differences at baseline in the BP180 ELISA indexes between patients with and without relapse in their retrospective analysis. The significance difference of BP180 ELISA index found in our study might result from proper dilution of serum for ELISA. ELISA must be performed using appropriate serum dilutions to demonstrate linear correlations between antibody concentration and index value. To be precise, with regard to the ELISA supplied from MBL Co., serum samples whose index is >100 must be tested with further dilutions (18). We performed further dilution in cases whose ELISA index exceeded 100. This is in accordance with previous reports showing that a high index of BP180 ELISA during the disease course or at cessation of therapy was predictive for relapse (13, 14). Our result suggested that an index value (53.09 U/ml) even at baseline was useful for prediction of clinical relapse. However, we also noted that some patients without relapse had a high index of BP180 ELISA at baseline and vice versa. It should be noted that a patient with a low BP180 ELISA index at baseline may possibly have relapse.

Bernard et al. (22). reported that a positive anti-BP180 detected by Western blotting was associated with poor prognosis. Although a high index of BP180 ELISA at baseline possibly is related to disease outcome, we did not analyze prognosis in our study.

Our results showed relatively higher serum IgE levels in the relapse group, which may indicate a risk for relapse, although there was no significance in this study. A previous report also showed no statistically significant correlation between serum IgE levels and extent of the disease (23). Interestingly, all three patients with negative reaction to both BP180 and BP230 ELISA at baseline were in the non-relapse group. Negative reaction to BP180 and BP230 ELISA might be a predictive sign of non-relapse. Overall, our study contained a small number of samples, although the frequency of relapse (34%) in our study was similar to that reported previously in Europe (12, 13) and Asia (24). Therefore, further study with larger sample sizes will be required.

## Conclusion

In this study, we reported a serological predictive marker at baseline for relapse in patients with BP. Our results indicated that higher index of BP180 ELISA (>53.09 U/mL) is a predictive marker for relapse at baseline. This may help clinicians treating BP patients in decision-making regarding duration and intensity of treatment.

## Ethics statement

This retrospective study was approved by the ethics committee of the Kurume University and was performed in accordance with the Declaration of Helsinki guidelines.

## Author contributions

HK and KT: contributed to study concept and design; HK: wrote the manuscript; HK, NI, and CO: contributed to the acquisition, analysis, and interpretation of data; TN: supervised the study. All authors had full access to all data in the study and take responsibility for the integrity of the data and the accuracy of the data analysis.

### Conflict of interest statement

The authors declare that the research was conducted in the absence of any commercial or financial relationships that could be construed as a potential conflict of interest.

## Abbreviations

BMZ: basement membrane zone
BP: bullous pemphigoid
ELISA: enzyme-linked immunosorbent assay
IF: immunofluorescence
IVIG: high-dose intravenous immunoglobulins
NC16A: non-collagenous 16A.
